# Role of Ambler Position 104 in Defining Substrate Specificity in the KPC Family of β‑Lactamases

**DOI:** 10.1021/acsinfecdis.6c00071

**Published:** 2026-04-28

**Authors:** Lin Gao, Steven Marshall, Christopher R. Bethel, Andrea M. Hujer, Magdalena A. Taracila, Kristine M. Hujer, Shozeb Haider, Robert A. Bonomo

**Affiliations:** 1 371646 UCL School of Pharmacy, London WC1N 1AX, U.K.; 2 Research Service, 20083 Louis Stokes Cleveland Department of Veterans Affairs Medical Center, Cleveland, Ohio 44106-1702, United States; 3 Department of Medicine, Division of Infectious Diseases, 12304 Case Western Reserve University School of Medicine, Cleveland, Ohio 44106-1702, United States; 4 University of Tabuk, Tabuk 71491, Saudi Arabia; 5 Departments of Molecular Biology and Microbiology, Pharmacology, Biochemistry, Proteomics and Bioinformatics, 12304 Case Western Reserve University School of Medicine, Cleveland, Ohio 44106, United States; 6 CWRU-Cleveland VAMC Center for Antimicrobial Resistance and Epidemiology (Case VA CARES), Cleveland, Ohio 44106, United States

**Keywords:** carbapenemase, KPC, cefiderocol resistance, ceftazidime avibactam resistance, P104K, P104R

## Abstract

Carbapenem-resistant Gram-negative bacteria pose a critical clinical challenge, largely due to the dissemination of class A *Klebsiella pneumoniae* carbapenemases (KPCs). Residue 104 in the α3−α4 loop of KPC enzymes forms part of a hydrophobic node influencing active-site architecture and substrate specificity. We systematically evaluated 19 amino acid substitutions at position 104 in KPC-3 to determine their impact on β-lactam and β-lactam/β-lactamase inhibitor susceptibility, enzyme kinetics, and structural dynamics. The *E. coli* containing the P104K and P104R variants exhibited markedly increased resistance to ceftazidime, ceftazidime/avibactam, ceftazidime/relebactam, and cefiderocol, while carbapenem susceptibility remained largely unchanged. Steady-state kinetics confirmed enhanced hydrolysis of ceftazidime and cefiderocol by these variants. Molecular dynamics simulations and deep-learning analyses revealed that substitutions at position 104 alter W105 orientation, expand active-site volume, and increase hinge-loop flexibility, enabling accommodation of bulky substrates. These findings highlight the critical role of residue 104 in shaping substrate specificity and inhibitor susceptibility in KPC enzymes, with implications for antimicrobial therapy and resistance evolution.

## Graphical abstract

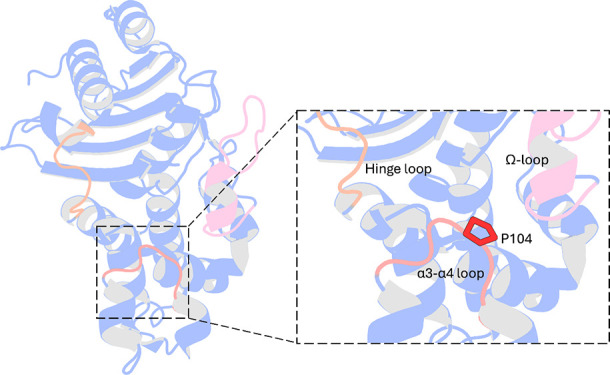

Carbapenem resistance among Gram-negative bacteria is one of our most urgent clinical and public health threats, especially when mediated by carbapenem-hydrolyzing β-lactamases.^1^ *Klebsiella pneumoniae* carbapenemases (KPC) are among the most widely disseminated class A serine β-lactamases that confer this phenotype. Previous investigations using adaptively sampled molecular dynamics (MD) simulations,^2^ demonstrated conserved regions of hydrophobic residues (nodes) within the KPC β-lactamase. These nodes are distributed and connected in a manner that forms hydrophobic networks, i.e., a series of nonpolar residues that interact via hydrophobic interactions. Markov State Models (MSM) and unsupervised deep learning permitted investigation that demonstrated the dynamics of these hydrophobic nodes can be used as a metastable relay of kinetic information within the core and are coupled with the catalytically permissive conformation of the active site environment. These observations support the hypothesis that these residues and networks are critical for structural integrity and β-lactam resistance. One of these important hydrophobic nodes is formed by the α3-α4 loop (amino acid residues 102–108) found in the distal flap of KPC. This loop plays a crucial role in the dynamics of W105, given that mutations at this position can abolish these interactions and significantly alter the free energy landscape of the W105 side chain in KPC.^2^

This previous research led us to fully explore the role P104 plays in this hydrophobic node of KPC and its effect on the resistance phenotype with regards to ceftazidime (CAZ), cefiderocol (FDC), imipenem (IMI), meropenem (MEM), ertapenem (ETP), and various β-lactam/β-lactamase inhibitor combinations ceftazidime/avibactam (CAZ/AVI), meropenem/vaborbactam (MEM/VAB), and imipenem/relebactam (IMI/REL) (Figure 1). Herein, we explored the functional consequences of substitutions at position 104. Our goal is to assess how amino acid changes at this residue influence substrate specificity.

**Figure 1:**
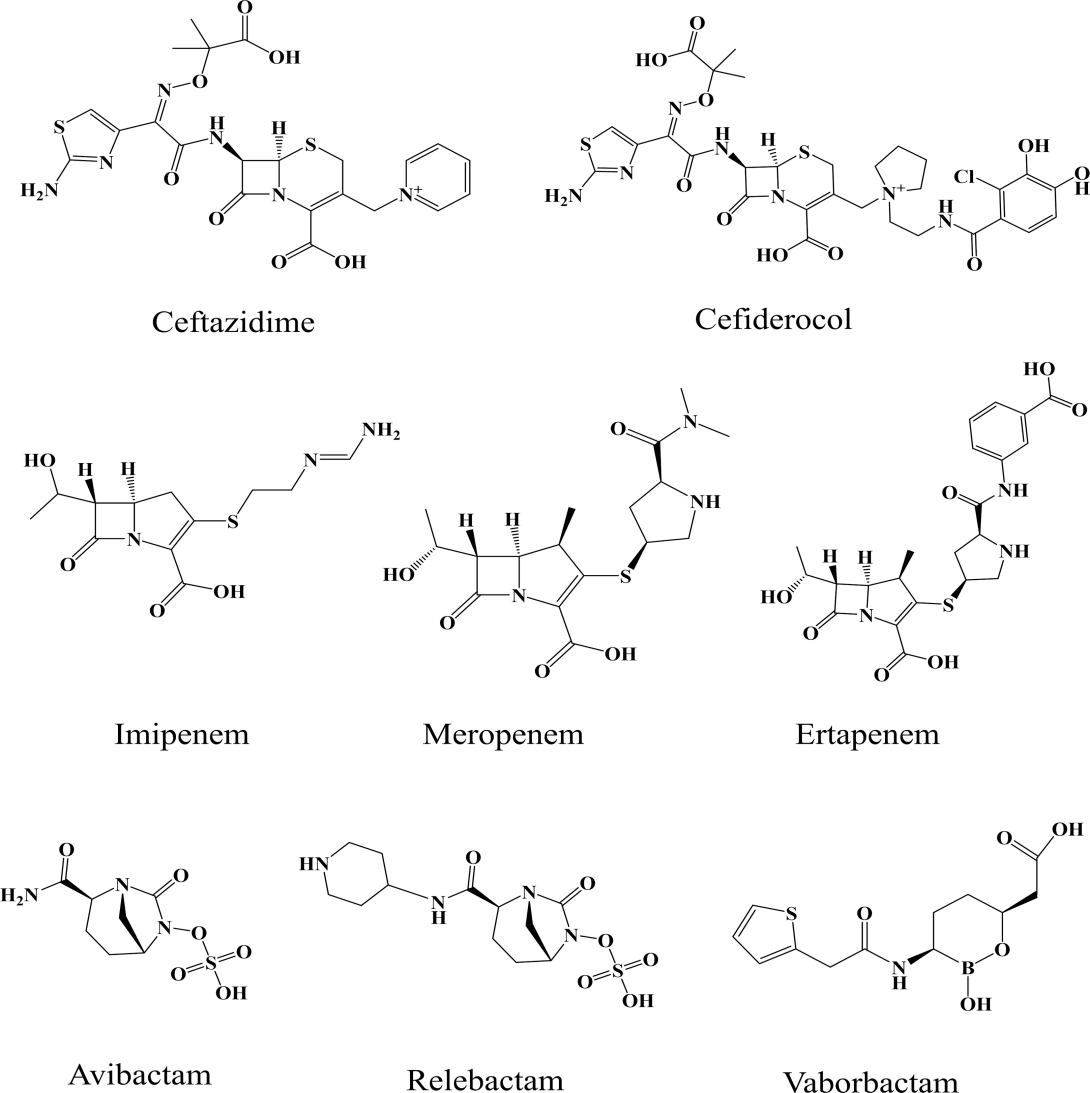
Structures of ceftazidime, cefiderocol, imipenem, meropenem, ertapenem, avibactam, relebactam, and vaborbactam.

Amino acid 104 is a proline (P) in KPC-3 carbapenemase. In other class A β-lactamases, variability is observed: in SHV the analogous 104 site is an aspartic acid (D), in CTX-M it is an asparagine (N), and in TEM and GES it is a glutamic acid (E). Structurally, the 104 site in class A enzymes is in the loop adjacent to the active site. This loop, comprised of amino acids 102–108 (α3-α4 loop), helps form the entrance to the active site, with the 104 forming a β-turn in that region.

Our investigations reveal that CAZ and CAZ/AVI susceptibility is altered as is resistance to FDC. In sharp contrast, very little change is seen with susceptibility to imipenem (IMI) and meropenem (MEM). The establishment of FDC and CAZ/AVI resistance along with the preservation of carbapenem resistance portends a very worrisome phenotype in KPC β-lactamases.

As substitutions at the 104 position occur clinically,^3–7^ we characterized this emerging phenotype that has significant consequences in the choice of antimicrobial therapy and in the understanding of the evolution of substrate specificity in class A β-lactamases.

## Results and Discussion

### Antibiotic Susceptibility

As seen in Table 1, a significant increase in CAZ MICs was observed for several of the *E. coli* containing KPC-3 variants, with the highest being a 3-fold increase (MIC = 4096 μg/mL) for P104H, P104K, and P104R when compared with KPC-3 WT (MIC = 512 μg/mL). A corresponding increase in MICs was detected for combinations with REL and AVI. CAZ/AVI MICs increased from 4 μg/mL (KPC-3 WT) to 16 μg/mL (P104K, P104R). CAZ/REL MICs increased as well from 8 μg/mL (KPC-3 WT) to 64 μg/mL (P104K, P104R). These same two substitutions showed significantly increased FDC MICs (1 μg/mL) when compared to KPC-3 WT (0.06 μg/mL) and came with a concomitant preservation of carbapenem resistance. The clinical implication is that bacteria with the P104K and P104R KPC-3 substitutions make for difficult-to-treat organisms.

**Table 1:** Minimum Inhibitory Concentrations (MICs) of *E. coli* DH10B Strains

	MICs (μg/mL)									
	CAZ	CAZ/AVI	CAZ/REL	FDC	AMP	IMI	IMI/REL	MEM	MEM/VAB	ETP
**pHSG298 Control**	1	≤0.5	≤0.5	0.06	8	0.5	0.25	0.06	0.06	0.015
**KPC-3/pHSG298**	512	4	8	0.06	8192	8	0.5	4	0.06	2
**P104A**	1024	4	8	0.125	8192	8	1	4	0.06	4
**P104C**	1	0.5	1	0.06	8	0.5	0.5	0.06	0.06	0.015
**P104D**	256	2	2	0.06	4096	16	1	4	0.06	4
**P104E**	256	2	2	0.06	8192	8	1	4	0.06	4
**P104F**	256	2	4	0.125	4096	8	0.5	2	0.06	2
**P104G**	512	8	16	0.125	8192	8	0.5	4	0.06	4
**P104H**	4096	8	16	0.25	4096	8	1	2	0.06	2
**P104I**	1024	8	8	0.06	4096	8	1	1	0.06	4
**P104K**	**4096**	**16**	**64**	**1**	4096	16	1	2	0.06	4
**P104L**	1024	4	8	0.25	4096	8	1	2	0.06	4
**P104M**	1024	4	8	0.125	4096	8	1	2	0.06	4
**P104N**	1024	4	16	0.125	4096	8	0.5	2	0.06	4
**P104Q**	2048	4	8	0.125	4096	4	0.5	2	0.06	4
**P104R**	**4096**	**16**	**64**	**1**	4096	8	1	2	0.06	4
**P104S**	2048	4	16	0.125	4096	8	1	2	0.06	4
**P104T**	1024	4	8	0.125	4096	16	1	2	0.06	2
**P104V**	256	4	8	0.06	4096	8	1	1	0.06	2
**P104W**	256	2	4	0.06	2048	8	1	1	0.06	2
**P104Y**	2048	4	4	0.25	2048	8	0.5	2	0.06	2

MICs for the P104C variant were reduced to levels of the pHSG298 empty vector control for all β-lactams (BL) and β-lactam/β-lactam inhibitor (BLI) combinations tested. Only P104A, P104E, and P104G variants conferred resistance to ampicillin (AMP) equal to that of the wild type (WT) (8,192 μg/mL). For the other variant β-lactamases a decrease in AMP MICs (2048 to 4,096 μg/mL) was observed. Carbapenem resistance was little changed. IMI MICs were within one dilution of WT KPC-3 (MIC = 8 μg/mL) ranging from 4 μg/mL to 16 μg/mL. MEM MICs for the variants either remained the same (4 μg/mL) or were lower (1–2 μg/mL). Similarly, ETP MICs remained relatively unchanged (2–4 μg/mL). The combinations of IMI/REL and MEM/VAB retained efficacy in lowering MICs for all 104 variants as well as KPC-3 WT (IMI/REL MIC = 0.5–1 μg/mL; MEM/VAB MIC = 0.06 μg/mL). These could be effective combinations against such pathogens.

### Immunoblotting

To determine what effect accumulation levels of the variant β-lactamases may have on MIC results, immunoblotting was performed. As shown in Figure 2, most variant proteins were present at similar or lower levels compared to KPC-3 WT. Greater amounts of KPC protein were observed for variants P104G, P104K, and P104F while no protein detection was observed for P104C. This could be due to formation of a Cys-Cys bond within the P104C variant resulting in a misfolded or unstable protein that may be more easily degraded, leading to less detectable β-lactamase, or perhaps substitution of a Cys at the 104 site alters translational efficiency. Previous immunogenic mapping of KPC β-lactamase demonstrated that amino acid 104 is not part of any of the main epitopes recognized by the polyclonal anti-KPC IgG antibody. Therefore, apparent differences in protein accumulation levels are not due to differential recognition of the KPC-3 variants by the antibody.

**Figure 2:**
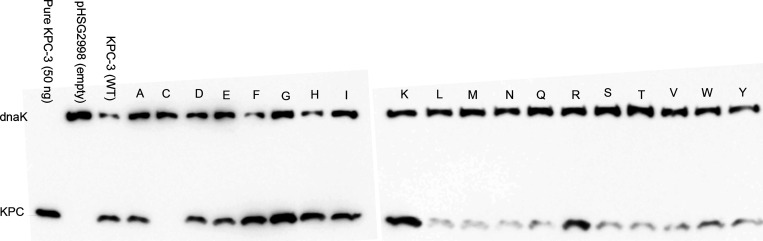
Immunoblot of KPC-3 (P104Xaa) variants using polyclonal anti-KPC-2 antibodies.

To further explore the increased CAZ and CAZ/AVI resistance observed in the MICs, purified KPC-3 (WT), KPC-3 P104K, and KPC-3 P104R were prepared and used for steady-state kinetics. KPC-3 P104R and KPC-3 P104K demonstrated higher CAZ hydrolysis (0.16 ± 0.02 s^–1^ μM^–1^ and 0.27 ± 0.02 s^–1^ μM^–1^, respectively), than KPC-3 (WT) (0.020 ± 0.002 s^–1^ μM^–1^) when using equal amounts of enzyme (Table 2), corroborating the higher CAZ MICs observed with the P104R and P104K variants. Similarly, KPC-3 P104R and KPC-3 P104K demonstrated higher FDC hydrolysis (0.019 ± 0.002 s^–1^ μM^–1^ and 0.016 ± 0.002 s^–1^ μM^–1^, respectively) than previously reported for KPC-3 (WT) (0.000250 ± 0.000006 s^–1^ μM^–1^)^8^ (Table 2), also corresponding to increased FDC MICs.

**Table 2:** Steady State Kinetic Parameters

	*K*_m_ (μM) or *K*_iapp_ (μM)			*k*_cat_ (s^–1^)			*k*_cat_/*K*_m_(μM^–1^ s^–1^)		
	KPC-3 (WT)	KPC-3 (P104K)	KPC-3 (P104R)	KPC-3 (WT)	KPC-3 (P104K)	KPC-3 (P104R)	KPC-3 (WT)	KPC-3 (P104K)	KPC-3 (P104R)
Ceftazidime	3504 ± 400	340 ± 40	595 ± 60	79 ± 8	95 ± 10	92 ± 10	0.020 ± 0.002	0.27 ± 0.03	0.16 ± 0.02
Cefiderocol	>500^a^	866 ± 65	424 ± 30	>0.13^a^	13.5 ± 1.2	8.4 ± 0.8	0.000250 ± 0.000006^a^	0.016 ± 0.002	0.019 ± 0.002
Avibactam	1.2 ± 0.1	1.6 ± 0.2	1.3 ± 0.2	–	–	–	–	–	–
Relebactam	1.1 ± 0.1	8.5 ± 0.9	10.4 ± 1.3	–	–	–	–	–	–

a Previously reported.^8^

Increased CAZ/AVI MICs were observed for the P104R (16 μg/mL) and P104K (16 μg/mL) variants vs KPC-3 (WT) (4 μg/mL). Using equal amounts of enzyme and nitrocefin as a reporter substrate, AVI *K*_i_s were measured (Table 2). Similar *K*_i_ values were obtained for KPC-3 (WT), KPC-3 P104R, and KPC-3 P104K (1.2 ± 0.1 μM, 1.3 ± 0.2 μM, and 1.6 ± 0.2 μM, respectively) indicating that the increased CAZ/AVI MICs are mainly due to increased CAZ resistance rather than AVI resistance. Increased CAZ/REL MICs were observed for the P104R (64 μg/mL) and P104K (64 μg/mL) variants vs KPC-3 (WT) (8 μg/mL). REL *K*_i_ determination revealed an increase for KPC-3 P104R and KPC-3 P104K (10.4 ± 1.3 μM and 8.5 ± 0.9 μM, respectively) vs KPC-3 (WT) (1.1 ± 0.1 μM) indicating that the drivers for increased CAZ/REL resistance include both an increase in CAZ and REL resistance.

### Electrospray Ionization Mass Spectrometry (ESI-MS)

Reaction courses (30 s, 1 min, and 15 min) showed no adduct formation of either ceftazidime (Figure S1) or FDC (Figure S2) with KPC-3 (WT), KPC-3 P104K, or KPC-3 P104R under the conditions. These results suggest that the CAZ and FDC are either hydrolyzed rapidly by the enzymes or are unable to bind to the enzymes.

### Structural Analysis

In computational studies, we focused on four systems including the KPC-3 wild type (WT), and the P104D, P104K, and P104R variant enzymes. In the P104D variant, the carboxylate side chain of residue D104 samples two major χ1 conformations which are trans (180°) and gauche (−)(−60°) (Figure 3A). When the D104 side chain adopts the trans (180°) conformation, the indole side chain of W105 shifts into a flip-out state characterized by a χ1 angle of trans (180°) (Figure 3B). This likely arises because the aspartate side chain occupies the space normally taken up by W105. This forces the indole side chain of W105 to rotate outward to avoid steric clashes. When D104 adopts the gauche (−)(−60°) conformation, the carboxylate side chain rotates away from the active site, becomes solvent-exposed potentially forming interactions with the surrounding water molecules (Figure 3C). This relieves steric hindrance and allows the W105 side chain to remain in the flip-in conformation. In contrast, in the KPC-3 WT, residue 104 is a proline. The χ1 density plot shows that proline occupies a single, relatively rigid conformation due to its constrained pyrrolidine ring (Figure 3A). Since proline does not create steric interference with W105, the side chain of W105 is packed against the proline ring and slightly favors the gauche (−)(−60°) χ1 conformation (Figure 3F). As a result, W105 maintains at a larger distance from residue T216 in the hinge loop (Figure 3G), corresponding to a stable flip-in conformation in which the indole ring points away from T216.

**Figure 3:**
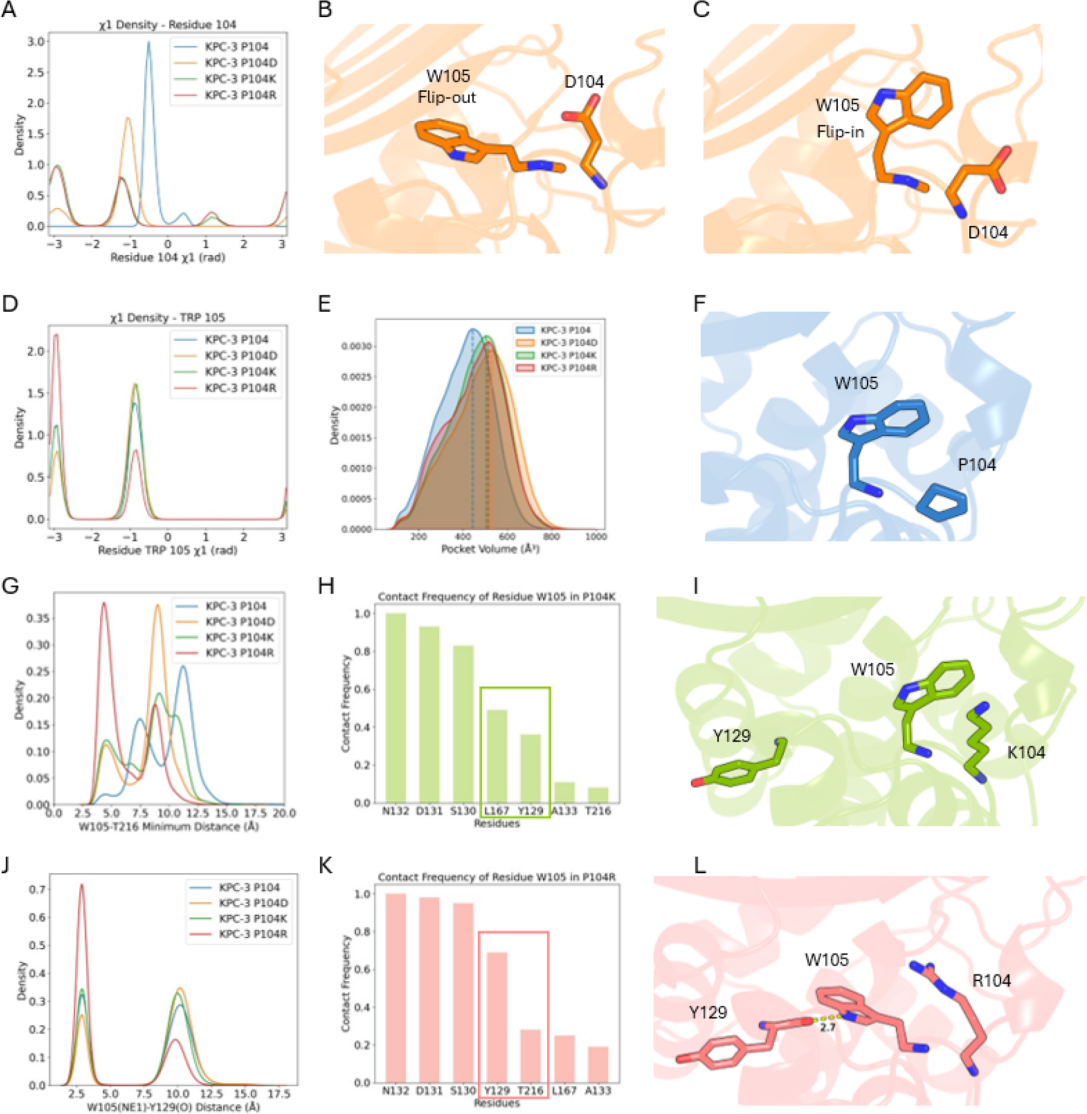
(A) χ1 density plots of residue 104 in KPC-3 WT and P104D, P104K, and P104R variants. Representative (B) flip-out and (C) flip-in conformations of W105 observed in the P104D variant. (D) χ1 density plot of W105 across all four systems. (E) Active site pocket volumes of the four protein systems. The modal pocket volumes are WT (P104) = 442.9 Å^3^, P104D = 529.4 Å^3^, P104K = 504.2 Å^3^, and P104R = 512.7 Å^3^. (F) Structural representation showing W105 packing over P104 in the KPC-3 WT. (G) Distribution of the minimum distance between W105 and T216 in all four variants. The contact frequency plots of W105 in (H) P104K and (K) P104R. Structural representations of interactions between W105 and residue 104 (K104 in panel I; R104 in panel L) and their spatial relationship with Y129. In the P104K variant, the side chain of W105 is positioned close to K104, whereas in P104R it resides further from R104 due to steric constraints. (J) Distance distribution between W105 and Y129.

Across all three variants, W105 can adopt conformations in which its side chain is positioned closer to T216 compared to the wild type. In the P104K and P104R variants, both lysine and arginine introduce positively charged side chains at position 104. These side chains strongly influence the preferred conformational state of W105. In the P104K variant, W105 shows low contact frequency with both L167 and Y129 (Figure 3H). In comparison, W105 in the P104R variant exhibits high contact frequency with Y129 but not with L167 (Figure 3K). In the P104R variant, the guanidinium group of R104 is strongly positively charged and capable of forming π-cation interactions with the indole ring of W105. However, its bulky and planar geometry allows it to occupy a broad, flat spatial region (Figure 3L). The guanidinium group could extend directly into the space occupied by W105 in the gauche (−)(−60°) state. This creates a substantial steric clash. As a result, W105 preferentially shifts into the trans (180°) conformation, rotating its indole ring outward and enabling it to form a hydrogen bond with the backbone of Y129 (Figure 3J). In contrast, in the P104K variant, the lysine side chain is narrower, less planar, and more flexible than arginine. This reduces steric interference with W105. Furthermore, the terminal NH_3_^+^ group of K104 can still engage in a π-cation interaction with the indole ring of W105 (Figure 3I). This may help in stabilizing the residue toward the active site. Consequently, W105 in the P104K variant shows a relatively higher preference for the gauche (−)(−60°) χ1 conformation.

In addition, the active site volumes were calculated for all four variants. Upon mutating proline to aspartic acid, lysine, or arginine, the active site volume increases in each case (Figure 3E). This expansion likely arises from the increased flexibility of the substituted side chains. Unlike proline, which is conformationally rigid, these residues possess multiple rotamers that allow their side chains to swing outward, thereby creating additional space within the active site. Such structural flexibility may facilitate improved accommodation of bulky substrates such as CAZ and FDC. Furthermore, the P104R variant exhibits a slightly larger active site volume than the P104K variant. This may result from W105 showing a greater tendency toward the flip-out conformation in P104R, which further enlarges the cavity.

### CVAE Embeddings

An unsupervised convolutional variational autoencoder (CVAE)-based deep learning^9^ was used to characterize the dynamic changes in the active site of KPC-3 and its variants (P104D, P104K, and P104R). The 22-dimensional latent space generated by the CVAE was further reduced to two dimensions using t-distributed Stochastic Neighbor Embedding (tSNE)^10^ to enable visualization of the overall conformational landscape. The tSNE projection reveals four distinct clusters, each representing a set of conformations sampled during the simulations.

From the cluster distributions, the WT system occupies a relatively compact region, with most conformations localized within cluster 3 (Figure 4A). In contrast, the P104K variant samples all four clusters with nearly equal distribution, indicating broader conformational heterogeneity. The P104D and P104R variants predominantly populate clusters 1, 2, and 4, further reflecting altered conformational preferences relative to WT. Overall, compared with the WT, all three variants exhibit greater conformational flexibility, whereas the WT remains comparatively more stable and structurally restricted.

**Figure 4:**
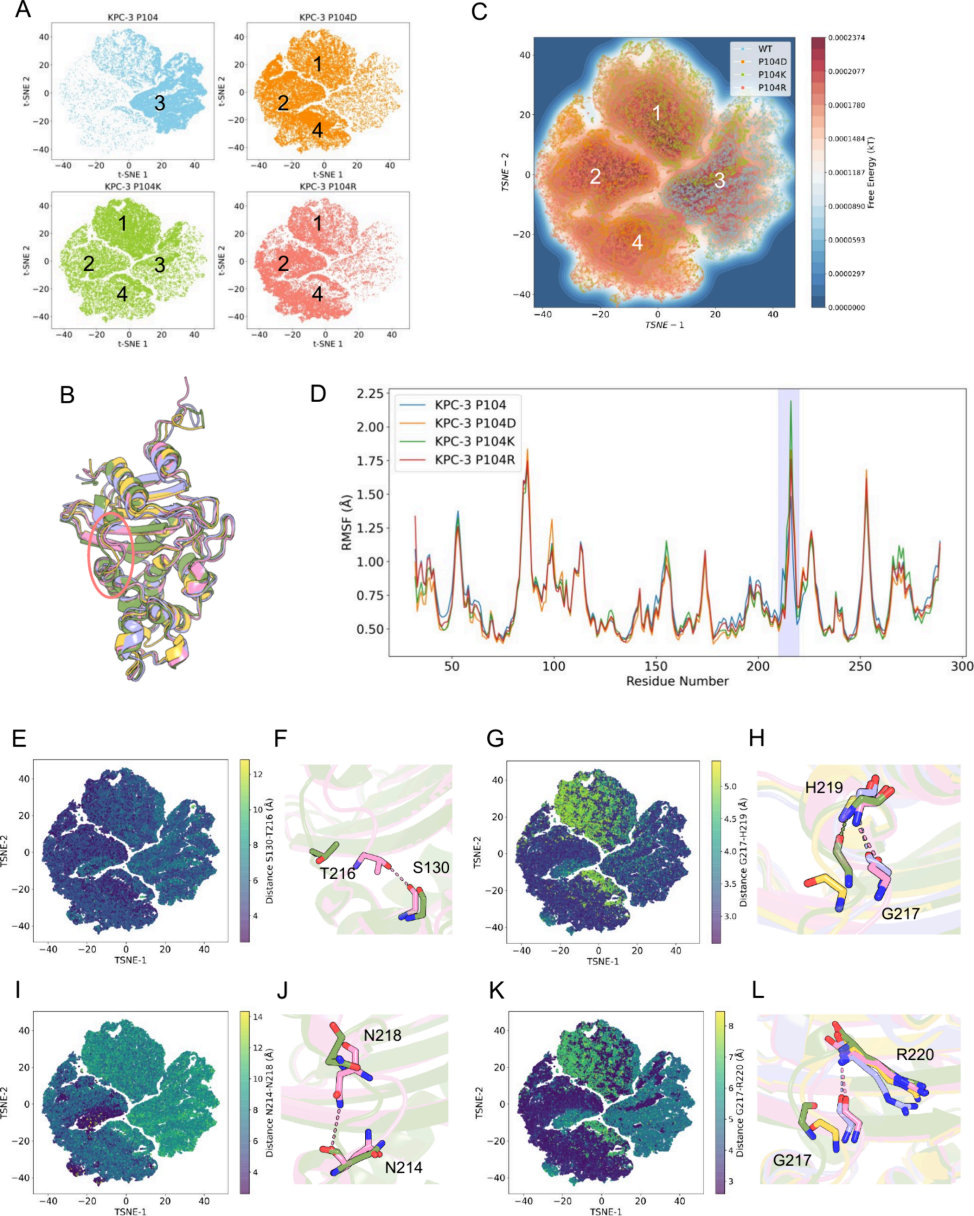
(A) CVAE-learned features of high-dimensional structural data visualized using a 2D tSNE projection for all four systems: KPC-3 WT (light blue), variants P104D (orange), P104K (green) and P104R (red). (B) Representative structures of the four conformational clusters: cluster 1 (yellow), cluster 2 (pink), cluster 3 (green), and cluster 4 (purple). The hinge region which exhibits structural variation among clusters is highlighted. (C) The free energy landscape of all 4 systems. (D) RMSF profiles of the four systems, showing substantial differences in flexibility within the highlighted hinge loop region. The tSNE projection colored according to the hydrogen bond distances for residue pairs (E) S130-T216, (G) G217-H219, (I) N214–N218 and (K) G217-G220. Structural representations of the corresponding residue pairs that may or may not participate in hydrogen bonding are shown for (F) S130-T216, (H) G217-H219, (J) N214–N218 and (L) G217-G220. Cluster colors correspond to those in panel B (yellow = cluster 1; pink = cluster 2; green = cluster 3; purple = cluster 4).

By extracting representative conformations from the free energy minima of each cluster (Figure 4C), it becomes clear that the hinge loop plays a central role in driving the dynamic differences among the four variants. The hinge loop undergoes substantial structural rearrangements (Figure 4B), particularly in its pattern of hydrogen bond interactions.

Clusters 2 and 4 exhibit a more compressed hinge loop conformation, with the loop oriented toward the active site. This inward movement is supported by the presence of a stabilizing hydrogen bond between S130 and T216 (Figure 4E,F), which is consistently observed in both clusters. In contrast, this interaction is absent in clusters 1 and 3, especially in cluster 3, which displays the most expanded hinge loop among all clusters. Additional hydrogen bonds further contribute to hinge loop compression in clusters 2 and 4. Residues N214 and N218, both within the hinge loop, form a stabilizing hydrogen bond that may shorten and draw the loop inward (Figure 4I,J). Interactions between G217 and R220, as well as a backbone hydrogen bond between G217 and H219, also promote a more compact hinge loop geometry (Figure 4G,H,K,L). These interactions collectively create a network of stabilizing contacts that bias the loop toward a closed, active-site-facing configuration. In contrast, these hydrogen bond interactions are largely absent in clusters 1 and 3. The interacting residues are positioned further apart, preventing the formation of stabilizing contacts seen in clusters 2 and 4. Consequently, the hinge loop in clusters 1 and 3 adopts a more expanded, outward-facing conformation.

Evaluation of RMSF profiles across the four systems (Figure 4D) further supports these observations. The hinge loop region displays notable differences in flexibility, consistent with the CVAE results showing that hinge loop rearrangements dominate the conformational variability among the clusters. In contrast, the Ω-loop exhibits minimal variation in flexibility across systems, suggesting that mutations at position 104 may have little structural impact on Ω-loop dynamics.

Overall, the hinge loop is the most flexible structural element in these systems and likely plays an important role in modulating the hydrolytic efficiency of KPC enzymes. The WT enzyme displays a more stable hinge loop conformation, while mutations at position 104 introduce increased dynamics and sample a broader conformational landscape. The conformations represented in clusters 1, 2, and 4 with their enlarged or reoriented hinge loops may contribute to the increased catalytic efficiency toward bulky substrates such as CAZ and FDC in the P104R and P104K variants. Among the variants, P104K displays the broadest distribution across the four CVAE clusters, reflecting multiple hinge loop conformations. This conformational diversity suggests that the P104K variant is particularly capable of adapting to substrates of varying size and shape, providing a structural basis for its expanded substrate profile.

## Discussion and Conclusions

Our results demonstrated greatly increased MICs against CAZ for the *E. coli* containing P104R and P104K KPC-3 variants, with concomitant increases in resistance to FDC, CAZ/AVI, and CAZ/REL. However, this increase in resistance was not observed for IMI, IMI/REL, MEM, and MEM/VAB. Previous work in our lab and others have demonstrated that the substitution in class A β-lactamases, e.g., of E104 K in TEM brought about a 4-fold increase in MIC and a 50-fold increase in catalytic efficiency with regards to CAZ.^11^ Looking specifically at the naturally occurring P104R (KPC-4, −5, and −10) and P104L (KPC-11) substitutions in a KPC-2 background, *E. coli* MICs demonstrated that the P104R substitution resulted in a 5-fold increase in resistance to CAZ, while the P104L substitution did not bring about a change in CAZ MICs.^4^ Even though WT KPC-2 hydrolyzes CAZ poorly, the P104R substitution brought about an 11-fold increase in catalytic efficiency for CAZ hydrolysis.^4^ It is interesting to note that with this increased catalytic efficiency for CAZ came a concomitant loss of enzyme stability (lower *T*_m_ values for the substituted enzymes). However, these substitutions did not significantly affect carbapenem MICs or hydrolysis.^4^

With regards to the 104 site and inhibitor susceptibility, for REL inhibition, KPC-4 (P104R/V240G) has a 3-to-4-fold higher IC_50_ than KPC-3 (260 nM vs 910 nM, respectively), and a 3-fold higher *K*_i app_.^11^ Whereas for AVI, KPC-4 has a 3-fold lower IC_50_ (9.3 nM) when compared to KPC-3 (29 nM).

The α3-α4 loop on which residue 104 is present, defines part of the active site of class A β-lactamases and is important in CAZ hydrolyzing ability in TEM β-lactamase. In TEM β-lactamase, mutation of E104 to lysine has been reported to increase CAZ MICs and catalytic efficiency, highlighting the functional importance of this position.^12,13^ Additionally, mutations at residue 104 in KPC enzymes influence the conformational preference of W105. Previous work has shown that P104R affects the positioning of W105 in KPC-4,^14^ and our results further support the strong coupling between residue 104 and W105 dynamics. W105 sits at the entry of the β-lactamase active site and is a key determinant of substrate and inhibitor specificity.^15^ Its large hydrophobic indole ring participates in substrate binding and helps stabilize β-lactam antibiotics within the catalytic pocket. Thus, subtle changes in the orientation of W105 may substantially alter enzymatic activity.

In the P104R variant, the combination of R104 and W105 introduces additional hydrophobic contacts and establishes a more favorable interaction surface for bulky substrates such as CAZ. The guanidinium group of R104 may force W105 toward the flip-out conformation, thereby enlarging the active site cavity. This expanded pocket is better suited to accommodate CAZ and form hydrophobic interaction with the dihydrothiazine ring of CAZ.^14^ Furthermore, the positively charged guanidinium group is well positioned to form stabilizing hydrogen bonds or electrostatic interactions with the carboxylate group of CAZ.^14^ This could potentially enhance substrate affinity and turnover. These combined effects may provide a structural basis for the increased catalytic efficiency and elevated CAZ MICs observed for the P104R variant.

A similar rationale may apply to P104K. Although lysine is less planar and less bulky than arginine, its positively charged terminal amino group can interact with incoming substrates and stabilize β-lactam binding. K104 also affects W105 orientation, albeit with a slightly weaker effect than R104, contributing to a more permissive and flexible active site. This correlates well with the experimentally observed increases in CAZ and FDC hydrolytic activity in both P104K and P104R.

The hinge loop which includes helix α11 lies opposite to the Ω-loop, also contributes to these functional differences. This region surrounds the active site and is structurally connected to both the allosteric pocket and the catalytic core.^16^ Consequently, its movement could influence the size, shape, and orientation of the active site cavity. Our results show that the hinge loop in the mutants adopts multiple conformations, particularly those represented in CVAE clusters 1, 2, and 4, and forms specific hydrogen bond networks that promote either compact or expanded loop states.

Mutations at residue 104 increase hinge loop flexibility compared to the WT enzyme. This enhanced plasticity may allow the active site to remodel dynamically in response to substrates of different sizes. In particular, the P104K and P104R variants sample conformations that enlarge the pocket or shift key residues such as W105 into orientations favorable for binding bulky β-lactams. These structural changes may provide a mechanistic explanation for their broadened substrate profiles and increased hydrolysis of CAZ and FDC.

## Materials and Methods

### Bacterial Strains

KPC-3 clones containing the native promoter and all 20 amino acid variants at position 104 were synthesized (Genscript Biotech Corp., Piscataway, NJ, USA) into a uniform vector (pHSG-298) and codon-optimized for *E. coli* DH10B for use in antibiotic susceptibility assays.

### Minimum Inhibitory Concentration (MIC) Measurements

MICs were determined by agar dilution as recommended by Clinical Laboratory Standards Institute (CLSI).^17^ Inhibitor concentrations were fixed at 4 μg/mL. All MICs were done in triplicate.

### Immunoblotting

Immunoblotting was used to evaluate KPC-3 WT and P104Xaa variant protein levels and performed as previously described.^15^ Five mL cultures of *E. coli* DH10B cells containing pHSG-298 phagemids harboring WT and *bla*_KPC‑3_ variants were grown in Mueller-Hinton (MH) broth containing 50 μg/mL kanamycin at 37 °C to an optical density at 600 nm (OD_600_) of 0.8. 50 μL aliquots were pelleted and frozen overnight. Pellets were resuspended in 20 μL loading dye, separated by SDS-PAGE (10% acrylamide), and transferred to a polyvinylidene difluoride membrane (Novex, Life Technologies, Carlsbad, CA, USA) by electroblotting. After blocking for 1 h with 5% nonfat dry milk, the blot was incubated in 5% nonfat dry milk with 1 μg/mL anti-KPC-2 polyclonal antibody and 1/10,000 dilution of mouse anti-DnaK monoclonal Ab (Enzo Life Sciences) overnight at 4 °C. The membrane was washed four times, 15 min each, in Tris-buffered saline (pH 7.4) containing 0.1% Tween 20 and subsequently incubated in 5% nonfat dry milk with 1/10,000 dilution of protein G-horseradish peroxidase conjugate. After four additional washes, membranes were processed for exposure using the ECL kit (GE Healthcare) and Azure 300 (Azure Biosystems).

### β-Lactamase Purification

β-Lactamase enzymes were expressed and purified as described previously.^15^ Briefly, *E. coli* DH10B cells containing the *bla*_KPC‑3,_ *bla*_KPC‑3 P104R_, or *bla*_KPC‑3 P104K_ genes were grown overnight in SOB medium, harvested by centrifugation at 4 °C, and frozen at – 20 °C. After thawing, β-lactamase was liberated using stringent periplasmic fractionation with 40 μg/mL lysozyme (Sigma) and 1 mM EDTA, pH 7.8. Preparative isoelectric focusing was performed with the lysate in a Sephadex granulated gel (GE Healthcare, Piscataway, NJ) using ampholines in the pH range 3.5–10 and running the gel overnight at a constant power of 8W on a Multiphor II isoelectric focusing apparatus (GE Healthcare). Purity was assessed by 5% stacking, 12% resolving sodium dodecyl sulfate–polyacrylamide gel electrophoresis. β-Lactamase concentration was determined using the Bio-Rad (Hercules, CA) Bradford protein assay with bovine serum albumin standards. A second purification step was performed using size-exclusion chromatography with a Pharmacia ÄKTA Purifier system (fast protein liquid chromatography instrument) (Uppsala, Sweden).

### Steady-State Kinetics

Steady-state kinetic parameters were determined using an Agilent 8453 diode array spectrophotometer at room temperature. Assays were performed in 10 mM PBS at pH 7.4. Kinetic constants (*K*_m_ and *V*_max_) were determined using a nonlinear least-squares regression of the data to the Henri-Michaelis–Menten equation (eq 1) in Origin, where v is the velocity of the reaction, *V*_max_ is the theoretical maximum velocity, *K*_m_ is the Michaelis constant, and [S] is the substrate concentration:

$$v=V_{\max}\left[S\right]/\left(K_{m}+\left[S\right]\right)$$ 1

A direct competition assay was performed to determine the relative dissociation constant, *K*_i app_. A final concentration of 5x *K*_m_ of nitrocefin was used as the indicator substrate in the presence of nanomolar concentrations of the β-lactamase. The data were corrected to account for the affinity of nitrocefin (*K*_m_^NCF^) for the β-lactamase according to eq 2, where S is the concentration of substrate used:

$${K_{i\;\mathrm{app}}}^{\left(\mathrm{corrected}\right)}={K_{i\;\mathrm{app}}}^{\left(\mathrm{observed}\right)}/1+\left[S/\left({K_{m}}^{\mathrm{NCF}}\right)\right]$$ 2

### Mass Spectrometry (ESI-MS)

Five micrograms of β-lactamase were incubated with the substrate at a molar ratio of 1:25 (FDC, CAZ) in sterile 10 mM phosphate-buffered saline (PBS) at pH 7.4 for a total reaction volume of 20 μL (t = 30 s, 1 min, 15 min). Reactions were quenched with 1% acetonitrile and 0.1% formic acid. Samples were analyzed using Q-TOF Waters Synapt-G2-Si and a Waters Acquity ultrapressure liquid chromatography (UPLC) BEH C18 column (1.7-μm pore size; 2.1 by 50 mm). MassLynxV4.1 was used to deconvolute protein peaks. The tune settings for each data run were as follows: capillary voltage at 3.5 kV, sampling cone at 35, source offset at 35, source temperature of 100 °C, desolvation temperature of 500 °C, cone gas at 100 L/h, desolvation gas at 800 L/h, and nebulizer bar at 6.0. Mobile phase A was 0.1% formic acid–water. Mobile phase B was 0.1% formic acid–acetonitrile. The mass accuracy for this system is ± 5 Da.

### Structure Preparation

The crystal structure of KPC-3 WT (PDB # 6QWD) was obtained from the Protein Data Bank. Point mutations P104D, P104K, and P104R were generated using PyMol’s mutagenesis tool.^18^ Four systems were prepared following a high-throughput molecular dynamics (HTMD) workflow.^19^ Protein parameters were assigned using the Amberff14SB force field,^20,21^ and the systems were solvated in a cubic TIP3P water box^22^ with a distance of 9 Å between any solute atom and the box edge. Each system was further neutralized and brought to a physiological ionic strength by adding 150 mM NaCl.

### Adaptive Sampling Simulations

The electrostatic interaction distances were set to £ 9 Å, and long-range electrostatics were treated using the particle mesh Ewald summation.^23^ Each system was first energy minimized using 3,000 steps of steepest descent, followed by a 5 ns equilibration in the NPT ensemble at 1 atm using a Berendsen barostat.^24^ The temperature was maintained at 300 K with a Langevin thermostat.

For the production phase, MSM-based adaptive sampling simulations were performed using the ACEMD molecular dynamics engine^19,25^ in the NVT ensemble with a 4 fs integration time step. The adaptive sampling protocol consisted of iterative rounds of short, parallel MD simulations. To reduce redundant sampling and accelerate exploration, a Markov state model (MSM) was constructed after each round to discretize the conformational landscape and estimate the free energy distribution from the stationary probabilities. Subsequent simulations were initiated from structures belonging to low free energy states. In this setup, the MetricSelfDistance function was used to monitor the number of native Cα-Cα contacts across all residues, which served as the structural metric for MSM construction. The exploration value of 0.01 and a goal function of 0.3 was used. Each adaptive sampling round comprised four parallel simulations of 100 ns each and continued until the total accumulated simulation time exceeded 30 μs. Trajectories were saved every 0.1 ns.

### Structural Analysis

All trajectories of KPC-3 WT and its mutants were aligned to their respective crystal structures using MDAnalysis.^26,27^ Hydrogen bond distances between relevant residue pairs were also computed using MDAnalysis. Minimum inter-residue distances were evaluated with PyEMMA.^28^ MDTraj^29^ was employed to calculate root-mean-square fluctuations (RMSF) and χ1 dihedral angles. Residue contact frequencies were obtained using MDciao.^30^ Structural visualization of all four systems was performed in PyMOL,^18^ and binding pocket volumes were quantified using MDpocket.^31^

### Convolutional Variational Autoencoder (CVAE) Based Deep Learning

A CVAE-based unsupervised deep learning^9^ was employed to characterize the conformational dynamics of KPC-3 wild type and its P104D, P104K, and P104R variants. Pairwise distance maps were generated for all Cα atoms within the active site region, including the hinge loop, Ω-loop, α3-α4 loop, and the α3 and α4 helices. Every tenth frame from the MD trajectories was extracted to construct these distance matrices. Each system produced a distance matrix of size 46 × 46, and the resulting four arrays were concatenated into a single data set. The model was trained using an 80:20 train-validation split, a batch size of 750, and 100 training epochs. Latent space dimensionalities ranging from 3 to 30 were evaluated, with a 22-dimensional latent space ultimately selected for the CVAE architecture. Following training, the decoded latent embeddings were projected into two dimensions using t-distributed stochastic neighbor embedding (tSNE)^10^ to facilitate visualization. When combined with free energy landscape analysis, this representation enabled the identification of distinct conformational states and the isolation of energetically favored structural minima.

## Supplementary Material



## Notes

ESI-MS time courses of KPC-3 (WT), KPC-3 P104R, and KPC-3 P104K using ceftazidime (CAZ) (Figure S1); ESI-MS time courses of KPC-3 (WT), KPC-3 P104R, and KPC-3 P104K using cefiderocol (FDC) (Figure S2) (PDF)

## References

[1] Spellberg B., Bonomo R. A. (2016). Editorial Commentary: Ceftazidime-Avibactam and Carbapenem-Resistant
Enterobacteriaceae: “We’re Gonna Need a Bigger Boat.”. Clin Infect Dis.

[2] Galdadas I., Lovera S., Pérez-Hernández G., Barnes M. D., Healy J., Afsharikho H., Woodford N., Bonomo R. A., Gervasio F. L., Haider S. (2018). Defining the
Architecture of KPC-2 Carbapenemase: Identifying Allosteric Networks
to Fight Antibiotics Resistance.. Sci. Rep.

[3] Castanheira M., Doyle T. B., Kantro V., Mendes R. E., Shortridge D. (2020). Meropenem-Vaborbactam
Activity against Carbapenem-Resistant Enterobacterales Isolates Collected
in U.S. Hospitals during 2016 to 2018. Antimicrob.
Agents Chemother..

[4] Mehta S. C., Rice K., Palzkill T. (2015). Natural Variants of the KPC-2 Carbapenemase
Have Evolved Increased Catalytic Efficiency for Ceftazidime Hydrolysis
at the Cost of Enzyme Stability.. PLoS Pathog.

[5] Robledo I. E., Aquino E. E., Santé M. I., Santana J. L., Otero D. M., León C. F., Vázquez G. J. (2010). Detection of KPC in *Acinetobacter* spp.
in Puerto Rico.. Antimicrob. Agents Chemother..

[6] Wang D., Chen J., Yang L., Mou Y., Yang Y. (2014). Phenotypic
and Enzymatic Comparative Analysis of the KPC Variants, KPC-2 and
Its Recently Discovered Variant KPC-15.. PLoS
One.

[7] Wolter D. J., Kurpiel P. M., Woodford N., Palepou M.-F. I., Goering R. V., Hanson N. D. (2009). Phenotypic and Enzymatic Comparative
Analysis of the
Novel KPC Variant KPC-5 and Its Evolutionary Variants, KPC-2 and KPC-4.. Antimicrob. Agents Chemother..

[8] Birgy A., Nnabuife C., Palzkill T. (2024). The Mechanism
of Ceftazidime and
Cefiderocol Hydrolysis by D179Y Variants of KPC Carbapenemases Is
Similar and Involves the Formation of a Long-Lived Covalent Intermediate.. Antimicrob. Agents Chemother..

[9] Bhowmik D., Gao S., Young M. T., Ramanathan A. (2018). Deep Clustering of Protein Folding
Simulations.. BMC Bioinformatics.

[10] Van
der Maaten L., Hinton G. (2008). Visualizing Data Using T-SNE. J. Mach Learn Res..

[11] Tooke C. L., Hinchliffe P., Lang P. A., Mulholland A. J., Brem J., Schofield C. J., Spencer J. (2019). Molecular Basis of
Class A β-Lactamase Inhibition by Relebactam. Antimicrob. Agents Chemother..

[12] Petit A., Maveyraud L., Lenfant F., Samama J. P., Labia R., Masson J. M. (1995). Multiple Substitutions at Position 104 of Beta-Lactamase
TEM-1: Assessing the Role of This Residue in Substrate Specificity. Biochem. J..

[13] Sowek J. A., Singer S. B., Ohringer S., Malley M. F., Dougherty T. J., Gougoutas J. Z., Bush K. (1991). Substitution of Lysine at Position
104 or 240 of TEM-1pTZ18R Beta-Lactamase Enhances the Effect of Serine-164
Substitution on Hydrolysis or Affinity for Cephalosporins and the
Monobactam Aztreonam. Biochemistry.

[14] Tooke C. L., Hinchliffe P., Bonomo R. A., Schofield C. J., Mulholland A. J., Spencer J. (2021). Natural Variants Modify *Klebsiella
Pneumoniae* Carbapenemase (KPC) Acyl–Enzyme Conformational
Dynamics to Extend Antibiotic Resistance. J.
Biol. Chem..

[15] Papp-Wallace K. M., Taracila M., Wallace C. J., Hujer K. M., Bethel C. R., Hornick J. M., Bonomo R. A. (2010). Elucidating the Role of Trp105 in
the KPC-2 β-Lactamase. Protein Sci..

[16] Galdadas I., Qu S., Oliveira A. S. F., Olehnovics E., Mack A. R., Mojica M. F., Agarwal P. K., Tooke C. L., Gervasio F. L., Spencer J., Bonomo R. A., Mulholland A. J., Haider S. (2021). Allosteric Communication in Class
A β-Lactamases
Occurs via Cooperative Coupling of Loop Dynamics. eLife.

[17] Clinical and Laboratory Standards Institute Performance Standards for Antimicrobial Susceptibility Testing; Thitrty-Second Informational Supplement M100-S34, 2024.

[18] Schrödinger, LLC The PyMOL Molecular Graphics System, Version 1.8, 2015.

[19] Doerr S., Harvey M. J., Noé F., De Fabritiis G. (2016). HTMD: High-Throughput
Molecular Dynamics for Molecular Discovery. J. Chem. Theory Comput..

[20] Case D. A., Cheatham T. E., Darden T., Gohlke H., Luo R., Merz K. M., Onufriev A., Simmerling C., Wang B., Woods R. J. (2005). The Amber Biomolecular Simulation
Programs. J. Comput. Chem..

[21] Maier J. A., Martinez C., Kasavajhala K., Wickstrom L., Hauser K. E., Simmerling C. (2015). ff14SB: Improving the Accuracy of
Protein Side Chain and Backbone Parameters from ff99SB. J. Chem. Theory Comput..

[22] Price D. J., Brooks C. L. (2004). A Modified TIP3P Water Potential
for Simulation with Ewald Summation. J. Chem.
Phys..

[23] Essmann U., Perera L., Berkowitz M. L., Darden T., Lee H., Pedersen L. G. (1995). A Smooth Particle Mesh Ewald Method. J. Chem. Phys..

[24] Berendsen H. J. C., Postma J. P. M., van
Gunsteren W. F., DiNola A., Haak J. R. (1984). Molecular
Dynamics with Coupling to an External Bath. J. Chem. Phys..

[25] Harvey M. J., Giupponi G., Fabritiis G. D. (2009). ACEMD:
Accelerating Biomolecular
Dynamics in the Microsecond Time Scale. J. Chem.
Theory Comput..

[26] Michaud-Agrawal N., Denning E. J., Woolf T. B., Beckstein O. (2011). MDAnalysis:
A Toolkit for the Analysis of Molecular Dynamics Simulations. J. Comput. Chem..

[27] Gowers, R. ; Linke, M. ; Barnoud, J. ; Reddy, T. ; Melo, M. ; Seyler, S. ; Domański, J. ; Dotson, D. ; Buchoux, S. ; Kenney, I. ; Beckstein, O. MDAnalysis: A Python Package for the Rapid Analysis of Molecular Dynamics Simulations; Los Alamos National Laboratory (LANL): Austin, Texas, 2016; pp 98–105. 10.25080/Majora-629e541a-00e.

[28] Scherer M. K., Trendelkamp-Schroer B., Paul F., Pérez-Hernández G., Hoffmann M., Plattner N., Wehmeyer C., Prinz J.-H., Noé F. (2015). PyEMMA 2: A Software Package for Estimation, Validation,
and Analysis of Markov Models. J. Chem. Theory
Comput..

[29] McGibbon R. T., Beauchamp K. A., Harrigan M. P., Klein C., Swails J. M., Hernández C. X., Schwantes C. R., Wang L.-P., Lane T. J., Pande V. S. (2015). MDTraj: A Modern
Open Library for the Analysis of Molecular
Dynamics Trajectories. Biophys. J..

[30] Pérez-Hernández G., Hildebrand P. W. (2025). Mdciao: Accessible Analysis and Visualization of Molecular
Dynamics Simulation Data. PLoS Comput. Biol..

[31] Schmidtke P., Bidon-Chanal A., Luque F. J., Barril X. (2011). MDpocket:
Open-Source
Cavity Detection and Characterization on Molecular Dynamics Trajectories. Bioinformatics.

