# Supplementary material for: Role of Ambler Position 104 in Defining Substrate Specificity in the KPC Family of β‑Lactamases
Source: ACS Infect Dis. 2026 Apr 28;12(5):1717–26. doi: 10.1021/acsinfecdis.6c00071 (PMC13162249; doi:10.1021/acsinfecdis.6c00071)
Supplement: Supplementary file 1 [file id6c00071_si_001.pdf]

Supporting Information:

Role of Ambler Position 104 in Defining Substrate Specificity in the KPC Family of β-lactamases

Lin Gao[1†], Steven Marshall[2†], Christopher R. Bethel[2], Andrea M. Hujer[3], Magdalena A. Taracila[2,3], Kristine M. Hujer[3], Shozeb Haider[1,4], and Robert A. Bonomo*[2,3,5,6]

[1]UCL School of Pharmacy, London, UK; [2]Research Service, Louis Stokes Cleveland Department of Veterans Affairs Medical Center, Cleveland, Ohio, USA; [3]Department of Medicine, Division of Infectious Diseases, Case Western Reserve University School of Medicine, Cleveland, Ohio, USA; [4]University of Tabuk, Tabuk, Saudi Arabia; [5]Departments of Molecular Biology and Microbiology, Pharmacology, Biochemistry, Proteomics and Bioinformatics, Case Western Reserve University School of Medicine, Cleveland, Ohio, USA; [6]CWRU-Cleveland VAMC Center for Antimicrobial Resistance and Epidemiology (Case VA CARES), Cleveland, Ohio, USA.

***Correspondence to**:

Robert A. Bonomo, M.D. (robert.bonomo@va.gov)

[†], Equal Contributions

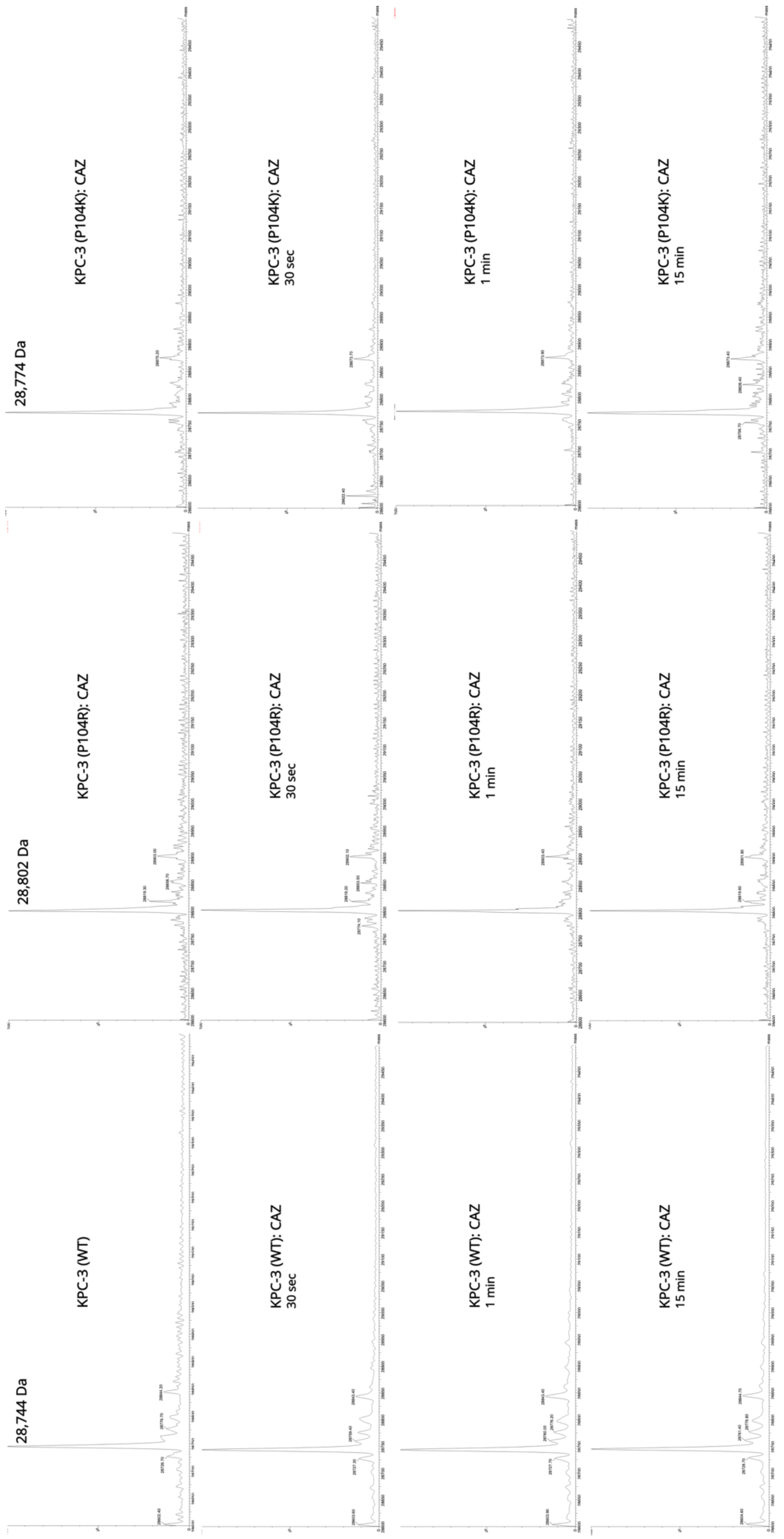

Figure S1. ESI-MS time courses of KPC-3 (WT), KPC-3 P104R, and KPC-3 P104K using ceftazidime (CAZ).

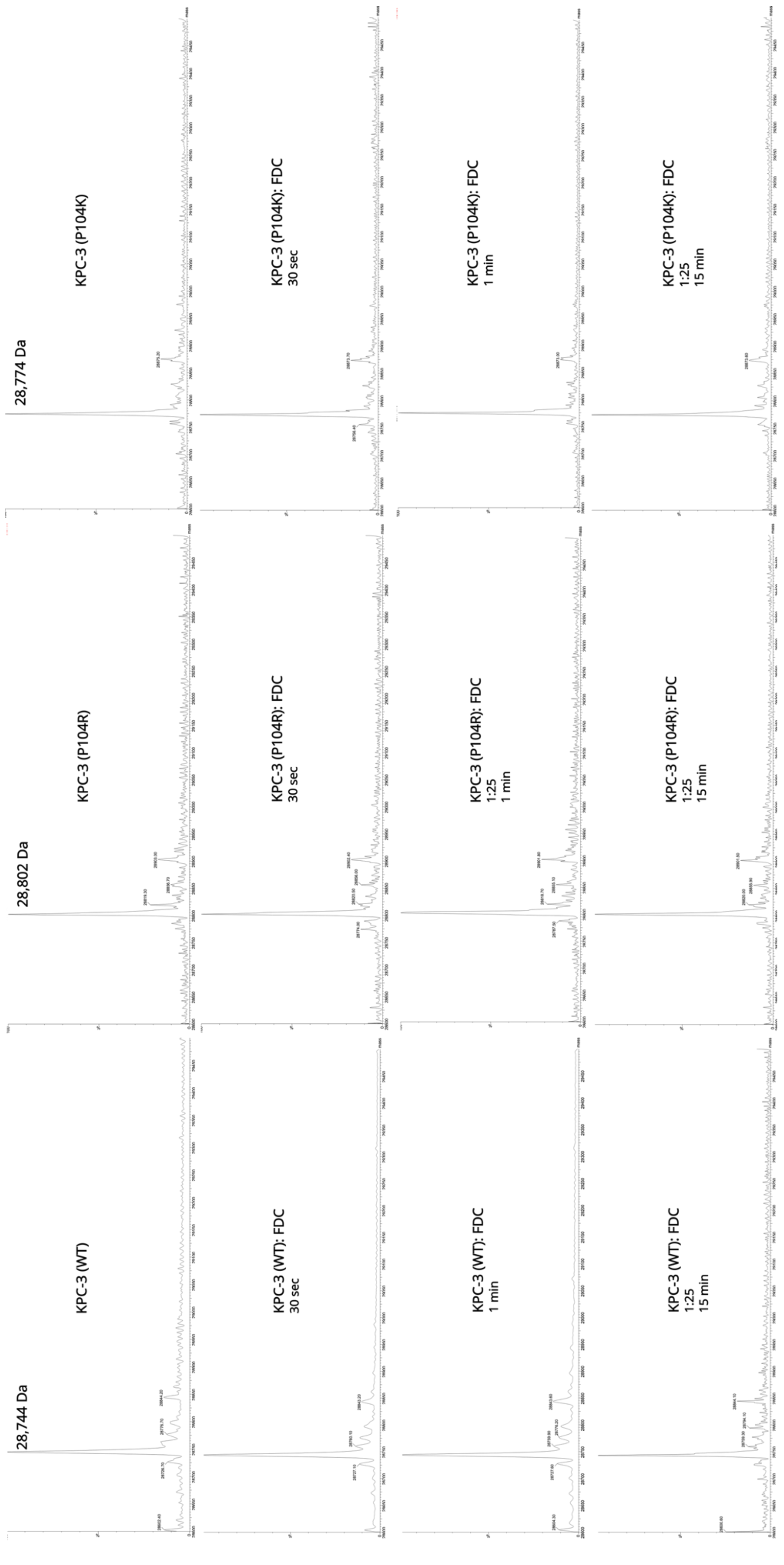

Figure S2. ESI-MS time courses of KPC-3 (WT), KPC-3 P104R, and KPC-3 P104K using cefiderocol (FDC).
